# Echocardiographic assessment of left atrioventricular coupling index: technical approaches and clinical implementation strategies

**DOI:** 10.3389/fcvm.2026.1833428

**Published:** 2026-07-03

**Authors:** Hengxiao Liu, Quan Li, Wenjie Han, Ziyao Shu, Peizhe Gao, Li Tian

**Affiliations:** 1 Henan University of Chinese Medicine, Zhengzhou, China; 2 Department of Ultrasound, The First Affiliated Hospital of Henan University of Chinese Medicine, Zhengzhou, China

**Keywords:** diastolic dysfunction, echocardiography, heart failure, left atrioventricular coupling index, technical standardization, three-dimensional echocardiography

## Abstract

**Background:**

The Left Atrioventricular Coupling Index (LACI) is an emerging echocardiographic parameter for assessing left atrial and ventricular diastolic function. While accumulating evidence supports its prognostic value, technical standardization for clinical implementation remains lacking.

**Methods:**

This narrative review compares four LACI measurement approaches: two-dimensional echocardiography (2DE), real-time three-dimensional echocardiography (RT-3DE), speckle-tracking echocardiography (STE), and artificial intelligence (AI)-assisted analysis. We evaluate technical characteristics, standardization challenges, and evidence gaps.

**Results:**

Two-dimensional ultrasound is optimal for screening; RT-3DE shows highest consistency with cardiovascular magnetic resonance for precise diagnosis, and 3DE-LACI has demonstrated independent prognostic value in cardiac amyloidosis. STE aids mechanistic exploration but requires algorithmic standardization. AI demonstrates potential but needs multicenter validation. Key limitations include absent cross-platform calibration, fragmented reference values, and lack of externally validated cutoffs.

**Conclusion:**

LACI is a promising marker of diastolic function with proven prognostic value; however, technical heterogeneity and inconsistent thresholds have hindered its clinical application. Future efforts should focus on cross-manufacturer standardization, the establishment of reference values for specific populations, and randomized controlled trials evaluating LACI as a guide for treatment.

## 1 Introduction

Although the left ventricular ejection fraction (LVEF) remains the most commonly used and classic indicator for assessing cardiac function in clinical practice, it lacks sufficient sensitivity in detecting abnormalities in cardiac diastolic function. Patients with heart failure with preserved ejection fraction (HFpEF) often exhibit normal LVEF values yet present with severe symptoms and poor prognosis. Traditional diastolic function parameters, such as the mitral inflow E/e′ ratio, left atrial volume index (LAVI), left atrial strain, and left ventricular global longitudinal strain (LV GLS), assess single-chamber function in isolation: E/e′ is load-dependent and technically demanding; LAVI quantifies atrial remodeling without accounting for ventricular compliance; LA strain reflects myocardial deformation but not chamber coupling; and LV GLS primarily evaluates systolic performance with limited sensitivity for early diastolic dysfunction (1). Because single-chamber indices cannot capture the atrial-ventricular coupling characteristics that characterize heart failure with preserved ejection fraction (HFpEF), a ratio-based index—the left atrial-ventricular coupling index (LACI)—has been developed. The introduction of LACI marks a shift in cardiac function assessment from single-chamber evaluation to inter-chamber interaction. Zornitzki et al. (2) provided an in-depth review elucidating the physiological significance of this index, establishing LACI as a sensitive indicator reflecting left ventricular compliance and left atrial load. LACI is defined as the ratio of left atrial end-diastolic volume (LAEDV) to left ventricular end-diastolic volume (LVEDV). When left ventricular diastolic function is preserved, rapid atrial blood emptying reduces LAEDV, resulting in a low LACI value. Conversely, impaired LV diastolic function diminishes suction force, preventing complete atrial emptying and elevating LACI. This mechanism explains why LACI abnormalities precede LVEF decline, conferring early warning value and motivating the search for clinically feasible measurement tools (3). Although cardiovascular magnetic resonance (CMR) first introduced the LACI concept (4), its clinical adoption remains limited due to expensive equipment, time-consuming examinations, and numerous contraindications (5). Echocardiography offers a promising alternative—real-time imaging, bedside accessibility, and no radiation (1)—but the diversity of technical pathways (2DE, RT-3DE, STE, AI) creates selection dilemmas. In addition, proprietary algorithms, operator dependency, and variations in acoustic windows all hinder the standardization process. Differences in LACI values across different technologies (6), insufficient validation in reference populations, and the lack of inter-device calibration methods all further hinder the implementation of standardization. Therefore, this paper examines the technical essentials and implementation challenges of LACI from an ultrasound specialist's perspective, aiming to address these gaps. Recent reviews have examined LACI from various perspectives, including multimodal approaches (7), prognosis (8), biventricular aspects (9), and pathophysiology (10); however, they have not systematically addressed the technical workflow specific to echocardiography—including protocol selection, quality control, and departmental implementation. This focused technology review compares four modalities—2D echocardiography, real-time 3D echocardiography, strain imaging, and AI-assisted analysis—in terms of technical characteristics, barriers to standardization, and clinical applicability, and proposes a practical implementation framework tailored for echocardiography departments.

Complementing the aforementioned reviews (Table 1), this paper does not aim for comprehensive coverage of the disease spectrum. Instead, it serves as a focused technical review addressing three specific questions: (1) Which echocardiographic technique (2DE, RT-3DE, STE, or AI-assisted) is best suited for specific clinical scenarios? (2)Why are existing normal LACI reference values difficult to apply directly, and what is needed to establish population-specific standards? (3)What quality control measures and phased steps are required to transition LACI from research to routine ultrasound reporting?

**Table 1:** Previously published LACI reviews vs. The Present Review: Core Differences.

Review (First Author/Year)	Journal	Core Theme	Technical Analysis Depth	Critical Appraisal	Implementation Strategy/Ultrasound-Specific Roadmap	Specialist Perspective
Poreba 2025	J Cardiovasc Dev Dis	Feasibility and clinical application of LACI assessment using multimodal imaging techniques	Systematic description	Briefly mentioned	None	None
Liu 2025	Front Cardiovasc Med	Prognostic value of LACI in cardiovascular diseases	Briefly mentioned	Briefly mentioned	None	None
Qin 2025	J Am Heart Assoc	Clinical utility of left and right atrioventricular coupling indices (LACI & RACI)	Systematic description	Systematic description	None	None
Afana 2025	Eur Heart J Cardiovasc Imaging	Comprehensive review of bi-atrioventricular coupling	Systematic description	Systematic analysis+bias assessment	None	Briefly mentioned
Present Review	—	Echocardiographic technical standardization and clinical implementation	In-depth comparison+scenario positioning	Systematic analysis + bias assessment	Phased in-depth implementation roadmap + practical tools	Ultrasound specialist-focused

## 2 Methods

This narrative review selectively synthesized the literature published between January 2021 and December 2025 regarding the assessment of LACI by echocardiography. This timeframe was chosen to capture the period following the establishment of a cardiovascular magnetic resonance-based prognostic benchmark in the MESA study (4), during which advanced echocardiographic techniques for LACI quantification (including real-time three-dimensional echocardiography, speckle-tracking strain analysis, and artificial intelligence-assisted segmentation) entered active clinical validation. Studies published prior to 2021 primarily utilized two-dimensional echocardiography or non-echocardiographic techniques and were therefore excluded from this focused technical review. Population stratification was predefined given the distinct physiological trajectories of cardiac chamber development: pediatric and adult data were analyzed separately throughout this review. Pediatric studies were included only to delineate age-specific reference patterns and developmental considerations, not for prognostic synthesis or clinical implementation recommendations, which focus exclusively on adult populations (≥18 years). Linden et al. (11) reported a characteristic U-shaped LACI trajectory in children (neonatal elevation, adolescent nadir, subsequent age-related increase), confirming that pediatric atrioventricular coupling dynamics differ fundamentally from adult patterns. Consequently, no pediatric data were pooled with adult cohorts in any prognostic or technical comparison.

### 2.1 Search strategy

PubMed, Embase, and Web of Science databases were searched. In PubMed, a combination of MeSH terms and free-text keywords was employed: MeSH terms included “Echocardiography,” “Heart Failure, Diastolic,” “Atrial Function, Left,” and “Artificial Intelligence”; free-text keywords included “left atrioventricular coupling index,” “LACI,” “two-dimensional echocardiography,” “three-dimensional echocardiography,” “real-time 3D echocardiography,” “speckle tracking,” and “strain imaging.” Boolean operators were applied as follows: (“left atrioventricular coupling index” OR “LACI”) AND (“echocardiography” OR “two-dimensional” OR “three-dimensional” OR “real-time 3D” OR “speckle tracking” OR “strain imaging” OR “artificial intelligence”). The search was restricted to English-language publications and human subjects, with no geographic restrictions. Embase and Web of Science were searched using analogous Emtree terms and keyword combinations, respectively, adapted to each database's indexing structure.

### 2.2 Review methodology

Given the high heterogeneity in study designs (cross-sectional, retrospective cohort, prospective validation), imaging modalities (2DE, RT-3DE, STE, CMR, CT), and outcome measures (diagnostic accuracy, prognostic HR/OR, technical consistency), a formal meta-analysis or pooled effect estimation was not feasible. This review did not follow the PRISMA statement for systematic reviews; instead, the search and synthesis were conducted with methodological transparency appropriate for a narrative review, and the reporting adheres to recommendations for expert-led technical reviews (12). Accordingly, we did not use a PRISMA flow diagram, risk-of-bias assessment tools, or study quality scoring, which are designed for systematic reviews.

### 2.3 Data synthesis

Due to substantial clinical and methodological heterogeneity across included studies—encompassing varying disease populations (HFpEF, HCM, AF, metabolic diseases), imaging modalities (2DE, RT-3DE, CMR, CT), and prognostic endpoints (all-cause mortality, heart failure hospitalization, atrial fibrillation incidence, stroke)—a formal meta-analysis with pooled effect estimates was not feasible. Instead, prognostic data were extracted and tabulated semi-quantitatively (reporting HR, OR, AUC, C-index, and 95% confidence intervals as presented in original studies) to facilitate cross-study comparison, while the overall synthesis remained qualitative and narrative. No new statistical analyses were performed on extracted data. The extracted prognostic data are summarized in Table 2.

**Table 2:** Summary of Key studies on LACI in cardiovascular diseases.

New number	First Author, Year	Disease/Population	Sample Size	Imaging Method	Key Findings (Effect Size, Cutoff)	Main Limitations
(3)	Dang 2025	HFpEF	60 HF (34 HFpEF) + 100 controls	2DE	LACI cutoff 33.07% for HFpEF diagnosis: AUC=0.951, sensitivity 97.1%, specificity 87.3%; Independent predictor (OR = 1.144)	Single-center, cross-sectional; small sample size; Vietnamese only
(4)	Pezel 2021	General population (MESA), free of CVD	4,124	CMR	LACI per 1 SD increase: AF: HR = 1.86, HF: HR = 1.50, CHD death: HR = 1.29, Hard CVD: HR = 1.23 (all *P* < 0.0001); Cutoff >25% for composite outcome	2D LA volume measurement; healthier cohort; no echocardiographic validation
(6)	Telders 2025	HFpEF	114	2DE vs CMR vs CT	Ultrasound-measured LACI showed high consistency with CMR; CT tended to overestimate LACI values	Conference abstract; preliminary data
(16)	Meng 2025	AL-CA	67	3DE (EchoPAC 204)	LACI per 1 unit: HR = 14.23 (univariable), adjusted HR = 10.58 for mortality; Cutoff ≥0.57; Added value to Mayo/Euro staging (all *p* < 0.001)	Single-center; short median follow-up (121 days); excluded AF patients
(19)	Zhu 2025	Paroxysmal AF post-ablation	100	RT-3DE	LACI: AUC=0.772 for recurrence; Combined with LAGLS: AUC=0.818	Single-center, retrospective; 1-year follow-up
(21)	Gao 2025	CKD stage 4−5 with/without T2DM	173	2DE + STE	LACI independent predictor of MACE (HR = 4.765); Cutoff 0.240: AUC=0.830; Diabetes independently associated with higher LACI (*β*=0.299)	Retrospective, single-center; short follow-up (median 21 months); small sample
(23)	Pezel 2023	Patients undergoing stress CMR (matched: normal vs abnormal)	2,134	CMR + AI (automated)	LACI per 0.1%: HR = 1.18 (HF hospitalization or CV death); LACI≥25%: HR = 3.27; Improved C-index from 0.72 to 0.76	Single-center, retrospective; no external validation
(24)	Zsarnoczay 2025	Severe AS undergoing TAVR	656	CT + AI	Preoperative LACI independent predictor of all-cause mortality after TAVR (C-index=0.72); outperformed traditional risk factors	Lack of multicenter prospective validation
(27)	Meucci 2022	HCM (no prior AF)	373	2DE	LACI≥40%: HR = 7.229 for new-onset AF; Better predictive value than LAVI or LAEF	Retrospective, single-center
(28)	Li 2024	Paroxysmal AF post-ablation	164	2DE	LACI: OR = 1.526 for recurrence; AUC=0.868	Single-center; 12-month follow-up only; Holter twice monthly may miss episodes
(29)	Fortuni 2024	Stable chronic HF (all LVEF ranges)	1,158 (derivation) + 242 (validation)	2DE	LACI per 1 SD: HR = 1.16 (all-cause death or HF hospitalization); Cutoff ≥0.32 for outcome; AUC=0.75 for moderate-severe DD	Retrospective; excluded AF; relatively low proportion of HFpEF (16%)
(31)	Wen 2025	HCM	206	CMR	LACI per 1%: HR = 1.054; Cutoff 40.09% for adverse outcomes; NRI=0.627, IDI=0.295	Single-center, retrospective; no external validation
(32)	De Raffele 2024	HCM	—	CMR	LACI independently associated with new-onset AF and ischemic stroke even without AF history	Conference abstract; limited detail
(33)	Demir 2026	HCM without AF	195	2DE	LACI > 61%: OR = 1.842 for ischemic cerebrovascular events; specificity 90.4%, sensitivity 62.5%	Single-center, retrospective; small number of events (*n* = 8)
(34)	Titichoatrattana 2025	HCM	—	—	LACI demonstrated good predictive value for adverse cardiac outcomes in HCM patients	Conference abstract; limited detail
(40)	Wu 2025	Severe AS undergoing TAVI	148	2DE	LACI≥28%: HR = 1.16 for MACE; Sensitivity 92%, specificity 56.9%; AUC=0.805	Single-center; moderate sample size; median follow-up only 13 months
(41)	Pezel 2022	General population (MESA)	1,911	CMR	LACI > 30%: HR = 1.91 for AF; *Δ*LACI > 1.5%/year: HR = 3.25 for AF; Improved C-index from 0.74 to 0.80	AF based on discharge codes; *Δ*LACI assumes linear change over 10 years
(45)	Vîjîiac 2024	DCM	121	3DE	LACI >20% is an independent predictor of MACE (C-index increased from 0.68 to 0.79)	Single-center; moderate sample size; follow-up period not specified
(46)	Meloni 2024	β-thalassemia	292	CMR	LACI >23.6% predicts cardiac complications: AUC=0.79, sensitivity 74.1%, specificity 75.8%	Cross-sectional design; limited sample size

## 3 Results

### 3.1 Technical characteristics of LACI measurement approaches

#### 3.1.1 Two-Dimensional echocardiography (2DE)

The two-plane modified Simpson method is a commonly used technical pathway for assessing left atrial volume via two-dimensional echocardiography. Two-dimensional echocardiography provides the most accessible approach for LACI screening. Large-scale normative studies in diverse populations [Arab (13) and Chinese cohorts (14)] have established age- and sex-stratified reference ranges, confirming LACI increases with age and is generally higher in females, particularly after age 50 (Table 2).The study further confirmed that LACI is independently associated with global longitudinal strain of the left ventricle and left atrial reserve strain, suggesting its potential value in identifying subclinical cardiac function alterations. The utility of two-dimensional LACI lies in screening and longitudinal follow-up. Therefore, clinical reports must ensure measurements are taken during the correct end-diastolic phase and clearly specify the technical approach (2D Simpson) and measurement method to avoid systematic errors due to phasing issues. Several key technical challenges, including systematic bias across different left atrial sizes and apical plane variability, remain to be addressed in future studies.Furthermore, the consistency of LACI measurements across different manufacturers’ 2D analysis software (Philips QLAB, GE EchoPAC, Siemens eSie) has not yet been studied or validated.

#### 3.1.2 Real-Time three-dimensional echocardiography (RT-3DE)

As the echocardiographic technique currently demonstrating highest consistency with CMR, real-time 3D ultrasound employs matrix probes to acquire full-volume data, enabling volume calculations without geometric assumptions. The accuracy of RT-3DE for left atrial volume quantification has been validated against CMR in multicenter studies (15). In light-chain cardiac amyloidosis (AL-CA), Meng et al. (16) demonstrated that 3DE-LACI provides independent prognostic value for all-cause mortality and incremental risk stratification beyond traditional clinical staging systems (Table 2). This study illustrates the prognostic utility of 3DE-LACI in restrictive cardiomyopathies, where reciprocal changes in LA and LV volumes amplify the index's sensitivity. However, the clinical adoption of 3D echocardiography still faces multiple technical bottlenecks. Regarding image quality, 3D probes typically operate at lower frequencies (1–4 MHz vs. 3–8 MHz) than 2D probes, resulting in reduced spatial resolution and frame rate limitations (20–40 fps vs.>60 fps), which compromises phase identification accuracy. Notably, the identification of end-diastole and end-systole becomes even more critical under low frame rate conditions (as in RT-3DE and CMR) and in the presence of ECG conduction disturbances, particularly complete left bundle branch block and right bundle branch block, which may introduce additional variability in QRS peak detection and ECG gating accuracy (17). Image quality degradation is more pronounced in obese patients, those with emphysema, or individuals with thoracic deformities. Additionally, in patients with significantly enlarged left atria, some portions of the atrial cavity may extend beyond the boundaries of the 3D pyramidal imaging sector, resulting in incomplete volume acquisition and potential underestimation of left atrial volume, as the entire left atrium may not be fully encompassed within the 3D imaging pyramid using currently available transducers (18). This technical limitation is particularly relevant in the context of left atrial enlargement, where chamber asymmetry and size may challenge accurate volumetric assessment even with three-dimensional echocardiography (15). In studies requiring accurate volume analysis, some patients are excluded due to insufficient image quality. Analysis timeliness is another challenge: semi-automated endocardial tracing requires 10–15 min per case, a time-consuming process that conflicts with routine ultrasound workflows. Zhu et al. (19) employed RT-3DE semi-automated analysis software to reduce single-case left atrial volume analysis to approximately 5 min, demonstrating automation-assisted potential. However, this study focused on clinical prognosis rather than software validation. Similar to 2D LACI, systematic variability exists in LACI measurements across different manufacturers’ 3D ultrasound systems (Philips, GE, Siemens, Canon). No calibration formula has been established, and there is no consensus on the optimal frame rate threshold for 3D full-volume sampling.

#### 3.1.3 Speckle tracking echocardiography (STE)

Speckle tracking echocardiography calculates left atrial myocardial strain by tracking the displacement of myocardial acoustic speckles, including reserve strain (LASr), conduit strain (LAScd), and contraction strain (LASct). STE and volumetric LACI reflect distinct pathophysiological mechanisms: LACI indicates left atrial ejection function, while strain reflects myocardial deformation capacity. Combined analysis distinguishes whether left atrial dysfunction stems from intrinsic myocardial strain impairment or load-induced LACI elevation. In patients with type 2 diabetes, LACI correlates with both left atrial and left ventricular stiffness indices, suggesting that abnormal glucose metabolism may impair atrioventricular coupling through dual mechanisms of myocardial fibrosis and altered diastolic function (20). In patients with CKD and diabetes, LACI serves as an independent predictor of major adverse cardiovascular events (MACE), with predictive efficacy surpassing that of left atrial volume index (LAVI) and left ventricular global longitudinal strain (LV GLS) (21) (Table 2). However, significant inter-vendor variability persists among different manufacturers’ STE algorithms, with strain values for the same patient showing variability up to 10%–15% (22). This prevents direct comparison of LACI-combined strain models derived from different platforms. Currently, conceptual consensus on LACI calculation via volumetric or strain methods remains elusive. A few exploratory studies have attempted simple combinations of LACI with STE parameters, but the physiological significance of such composite metrics remains inadequately validated. Normal reference values still rely on proprietary manufacturer databases, and large-scale multicenter studies are lacking.

#### 3.1.4 Artificial intelligence (AI)-assisted analysis

In the LACI field, artificial intelligence was first applied in CMR. Pezel et al. (23) developed a fully automated LACI analysis system based on deep learning, capable of segmenting left atrial and ventricular volumes within 1 s, demonstrating the feasibility of automated segmentation for complex cardiac structures in a large stress CMR cohort. This system showed robust prognostic stratification, with LACI incrementally improving cardiovascular risk prediction beyond traditional risk factors and established CMR parameters (Table 2). However, the model's generalizability remains uncertain given the lack of external multicenter validation. While one study (19) reported that automated 3D ultrasound analysis software reduced left atrial volume analysis time from 15 to 5 min, manual boundary correction remains necessary. The CT-based AI-LACI system developed by Zsarnoczay et al. (24) achieved fully automated preoperative risk stratification for TAVR, demonstrating AI's potential in risk assessment. However, clinical translation remains constrained by bottlenecks, including the lack of multicenter prospective validation. Currently, device independence has not been achieved; significant image texture variations exist across different echocardiography devices, and no cross-platform universal model is available, limiting the conduct of multicenter studies. AI models also face challenges in recognizing atrial fibrillation rhythms, typically trained on stable rhythms. When RR intervals become irregular, phase determination errors occur, necessitating specialized algorithms for correction.

**Figure 1:**
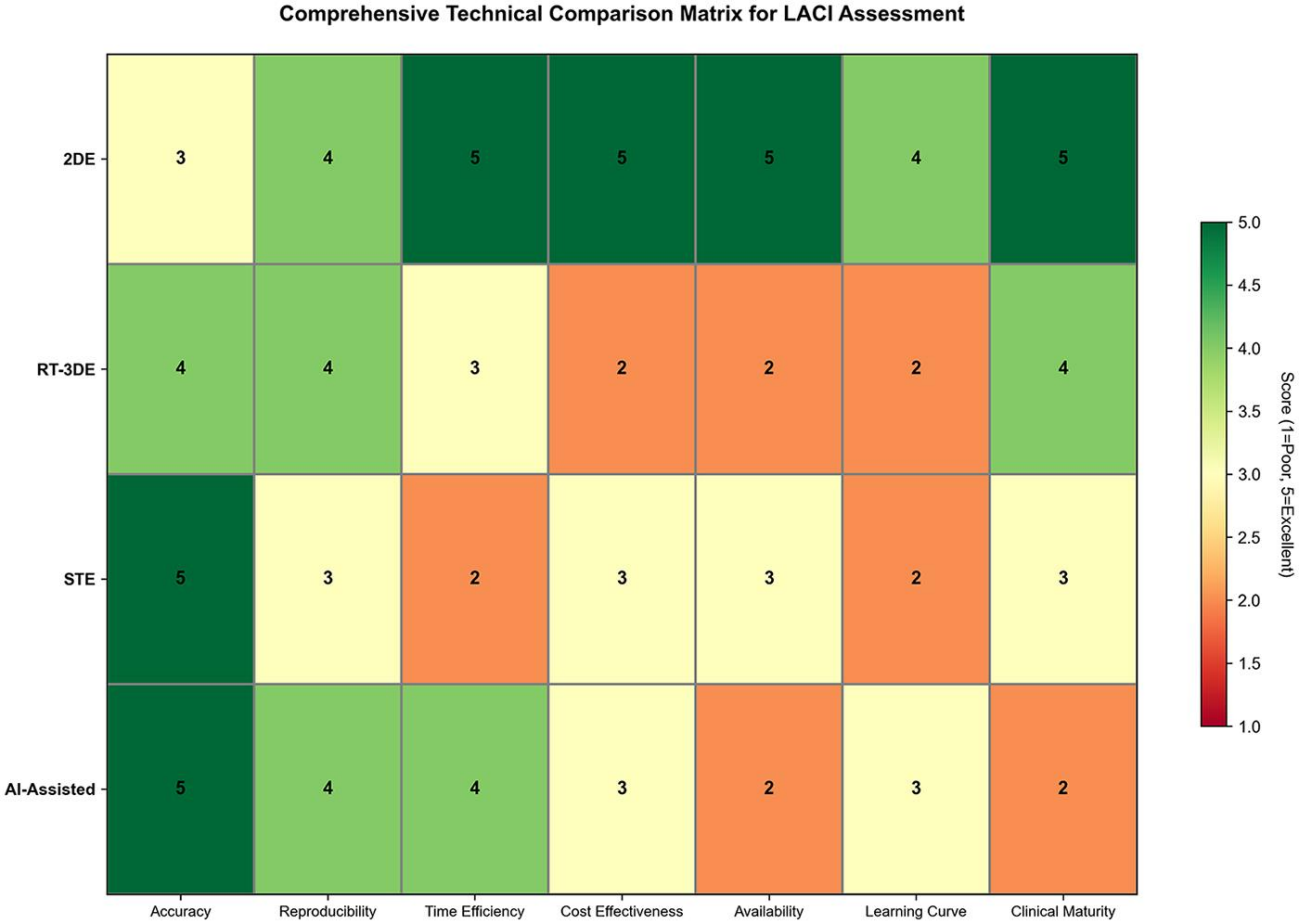
Performance matrix comparing four LACI measurement approaches (2DE, RT-3DE, STE, AI) across accuracy, time consumption, accessibility, and clinical applicability. 2DE is optimal for screening; RT-3DE shows highest accuracy (r = 0.88−0.94 vs. CMR); STE aids mechanistic exploration; AI requires multicenter validation.

Figure 1 provides a matrix-based comparison of the relative performance of the four technical pathways across dimensions such as accuracy, time consumption, and accessibility.

#### 3.1.5 Exercise stress testing and stress assessment

At rest, LACI measurements may fail to detect early or occult diastolic dysfunction. Exercise stress testing can assess functional reserve. Backhaus et al. (25) used CMR exercise testing and found that patients with normal resting LACI exhibited significant abnormalities during exercise. Furthermore, by establishing a normal response pattern for exercise-induced LACI, this study provides a basis for early intervention. Research (23) combining artificial intelligence with stress CMR offers a reference for automated analysis of ultrasound exercise-induced LACI. Exercise-induced LACI can identify occult diastolic dysfunction; however, in the field of ultrasound, research on exercise-induced LACI remains unexplored, presenting promising prospects for clinical translation. AI-based fully automated analysis methods validated in CMR provide reference for the ultrasound field.

### 3.2 Reference values and standardization challenges

#### 3.2.1 Existing reference values

Current normal LACI reference values primarily derive from large-scale two-dimensional echocardiography study Arab (13) and Chinese (14) populations, as well as from a multiethnic CMR cohort (4) (Table 2). These studies provide important clinical benchmarks but exhibit significant methodological and population biases. Significant ethnic differences exist in LACI distribution. Although the MESA cohort study (26) did not directly report ethnicity-specific LACI reference values, analysis of left atrial functional parameters revealed that Chinese Americans had the smallest left atrial volume and highest ejection fraction, while African Americans had the largest left atrial volume and poorest functional parameters. Since LACI represents the ratio of left atrial volume to left ventricular volume, its normal distribution also exhibits significant ethnic specificity. For Chinese populations, LACI values may be lower than those observed in African American populations. Pezel et al. (4) further confirmed that the prognostic value of LACI is consistent across ethnic groups, underscoring the necessity of establishing ethnicity-specific reference values. Accurate identification of abnormally elevated LACI and its associated clinical risks requires clear baseline values specific to each ethnic group. Age stratification differences also impact LACI's clinical translation. While existing studies predominantly use 10-year age brackets, the relationship between LACI and age may be nonlinear. As pediatric data were analyzed separately from adult cohorts (see Methods), the following developmental pattern is presented for reference completeness only and does not inform adult clinical cutoff recommendations. Linden et al. (11) observed a “U-shaped” trajectory in children, with LACI levels elevated in the neonatal period, reached a nadir during adolescence, and subsequently increased with age. Whether a similar inflection point exists in adults requires detailed stratified research. Furthermore, the interactive effects of gender, body surface area, and physical activity levels remain inadequately elucidated.

#### 3.2.2 Cutoff values

**Figure 2:**
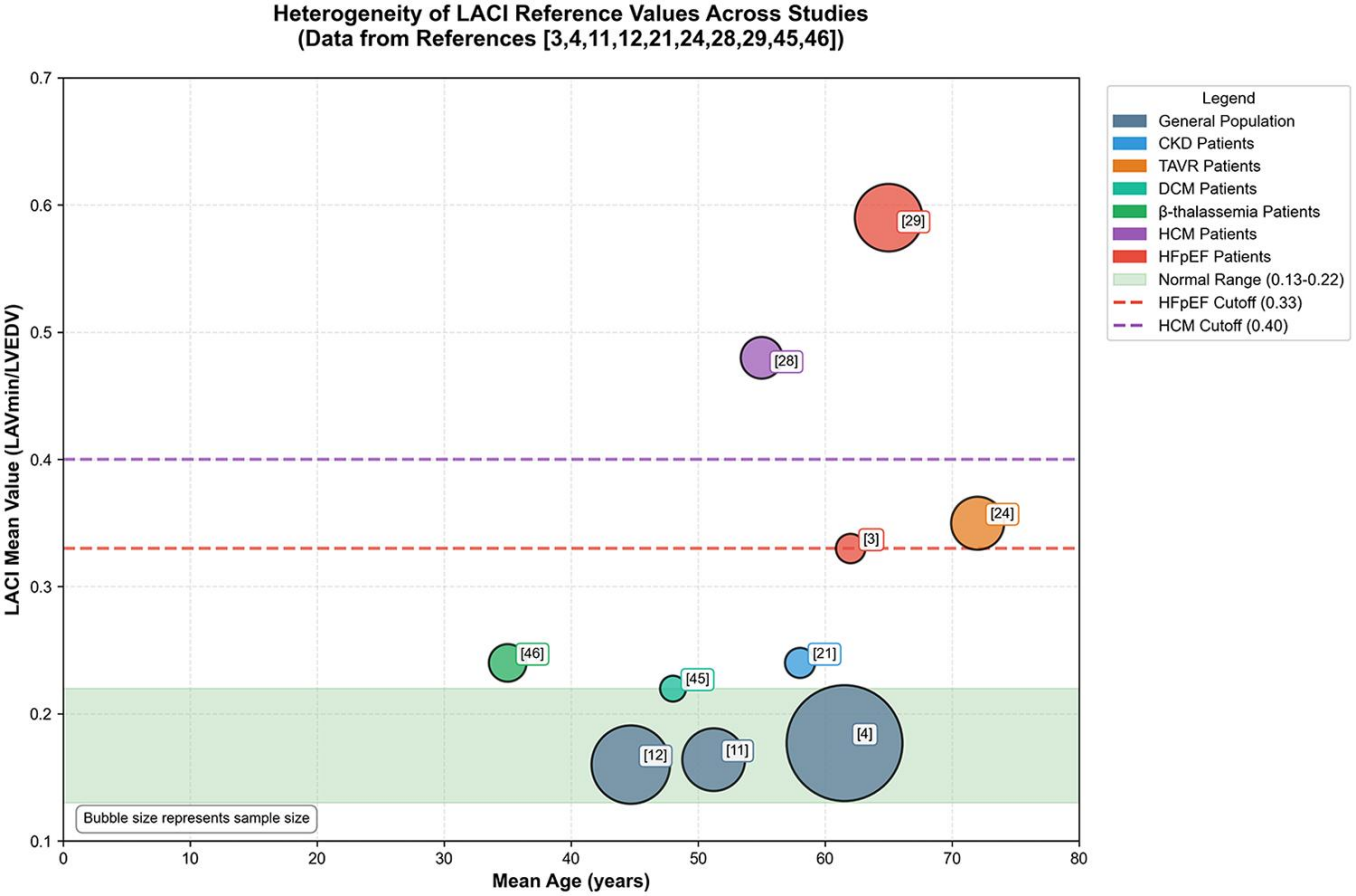
Bubble chart showing LACI reference value distribution across studies. Bubble size represents sample size; color indicates population type. Notable heterogeneity exists between healthy and diseased populations across ethnic groups.

The prognostic cutoff values for LACI exhibit significant disease heterogeneity. In HFpEF, LACI is markedly elevated compared with controls and demonstrates high diagnostic accuracy for identifying diastolic dysfunction (3). In HCM, LACI serves as a strong predictor of new-onset atrial fibrillation, independent of left atrial volume index and left ventricular outflow tract pressure gradient (27). Regarding AF ablation prognosis, preoperative LACI values represent an independent risk factor for postoperative recurrence (28) (Table 2).Caution remains warranted regarding the universal applicability of a single cutoff value. HFpEF patients with different etiologies—such as hypertensive, obese, or metabolic causes—may exhibit distinct patterns of left atrial remodeling. Applying a uniform cutoff could lead to misclassification. Furthermore, current cutoff values are largely based on single-center retrospective studies, lacking external validation and cost-effectiveness analysis. Figure 2 presents the distribution heterogeneity of LACI reference values across different studies using a bubble chart, where bubble size represents sample size and color indicates population type, revealing significant differences between diseased and healthy populations.

### 3.3 Clinical applications across disease Spectra

#### 3.3.1 Heart failure with preserved ejection fraction (HFpEF)

HFpEF represents the disease domain where LACI demonstrates the greatest clinical value. Initial studies (3) reported significantly elevated LACI values in HFpEF patients. Telders et al. (6) demonstrated in a conference abstract that ultrasound-measured LACI showed high consistency with CMR-measured LACI in a comparative study of 114 HFpEF patients, while cardiac CT tended to overestimate LACI values, providing cross-technology validation for ultrasound application. Fortuni et al. (29) demonstrated that LACI, as a novel diastolic function parameter, holds significant value in assessing heart failure prognosis and is independently associated with adverse outcomes in heart failure patients. Wang et al. (30) further emphasized LACI's substantial potential in identifying diastolic dysfunction, noting that its clinical implementation hinges on achieving technical standardization. Current LACI studies predominantly employ cross-sectional designs, reflecting only the prognostic value of single measurements.

#### 3.3.2 Hypertrophic cardiomyopathy (HCM)

HCM represents the disease with the most robust clinical evidence for LACI. In addition to its established value in predicting new-onset atrial fibrillation (27), LACI has been validated by CMR as an independent predictor of adverse outcomes, providing gold-standard evidence of its prognostic utility in this population (31) (Table 2).

More clinically significant is the absence of atrial fibrillation-related stroke. De Raffele et al. (32) found that even without a history of atrial fibrillation, patients with HCM and elevated LACI exhibited a significantly increased risk of ischemic stroke suggesting LACI may reflect thrombus-forming mechanisms such as left atrial blood stasis and endothelial dysfunction. Demir et al. (33) further confirmed in HCM patients without AF that those experiencing ischemic cerebrovascular events had significantly higher baseline LACI levels than event-free individuals. Multivariate analysis showed LACI was an independent predictor of ischemic events (Table 2). Titichoatrattana et al. (34) also found that LACI demonstrated good predictive value for adverse cardiac outcomes in HCM patients. De Raffele et al. (35) further reported differences in left atrial remodeling across left ventricular hypertrophy phenotypes.

#### 3.3.3 Metabolic diseases

Metabolic syndrome and diabetes represent emerging application areas for LACI. Research first demonstrated (20) that elevated LACI levels in type 2 diabetes patients precede traditional diastolic function indicators, offering early warning value. Shi et al. (36) employed CMR strain imaging to reveal that patients with diabetes and hypertension exhibited significantly higher LACI values than those with diabetes alone, independently associated with reduced left atrial functional reserve. This indicates synergistic detrimental effects between metabolic diseases and cardiovascular risk factors. Another study (21) in patients with CKD and diabetes also suggested that LACI holds considerable value for risk stratification of myocardial damage in metabolic diseases. Zhou et al.'s (37) CMR study confirmed that left atrial reserve and conduit function were already impaired in prediabetic patients, though LACI showed no significant difference compared to controls, indicating that LACI changes lag behind strain metrics. Metabolic-associated fatty liver disease (MAFLD) warrants equal attention. Huang et al. (38) found that among patients with metabolic syndrome, those with MAFLD exhibited significantly elevated LACI compared with both non-MAFLD metabolic syndrome patients and healthy controls, identifying MAFLD as an independent factor influencing LACI elevation. Furthermore, LACI showed negative correlations with both left atrial reserve and conduit function, suggesting MAFLD may cause atrioventricular decoupling by impairing left atrial function (Table 2). Hepatic metabolic dysfunction may influence cardiac function through systemic inflammation and oxidative stress pathways (38). Zhang et al. (39) further confirmed that patients with type 2 diabetes exhibit more pronounced atrioventricular coupling abnormalities when complicated by functional mitral regurgitation.

#### 3.3.4 Interventional treatment for structural heart disease

LACI also has applications in interventional settings. A pioneering study (24) using AI-automated LACI analysis via CT found that preoperative LACI was an independent predictor of all-cause mortality after TAVR (C-index=0.72), outperforming traditional risk factors. Wu et al. (40) further confirmed through a prospective echocardiographic study that preoperative LACI ≥ 28% was an independent risk factor for major adverse cardiovascular events at 1 year post-TAVR (HR = 1.16, 95% CI: 1.10–1.22). ROC analysis revealed this cutoff point exhibits high sensitivity and acceptable specificity (sensitivity 92%, specificity 56.9%). Kaplan–Meier curves confirm that patients with LACI ≥28% demonstrate poorer prognosis.

#### 3.3.5 Atrial fibrillation

The MESA study by Pezel et al. (41) established the long-term predictive value of LACI for atrial fibrillation occurrence. Through long-term CMR follow-up of multiethnic participants without prior cardiovascular disease, the study found that both baseline and serial LACI measurements were independently associated with incident AF. Moreover, dynamic LACI growth (*Δ*LACI) demonstrated stronger predictive power than single-timepoint assessment, with progressive LACI elevation conferring a markedly increased AF risk. Incorporating *Δ*LACI into the CHARGE-AF risk score significantly improved the model's discriminatory ability and reclassification performance (41) (Table 2).This finding indicates that tracking LACI trends more sensitively reflects left atrial myocardial disease progression than a single measurement. Karanikola et al. (42) further demonstrated that elevated LACI serves as a marker of atrial myocardial disease in AF patients, correlating with AF burden and recurrence risk. Other studies (19, 28) also confirmed LACI's efficacy in predicting recurrence after ablation. However, measuring LACI during atrial fibrillation remains challenging: irregular R-R intervals cause errors in single-cycle calculations, while averaging multiple cycles increases analysis time.

#### 3.3.6 Other cardiomyopathy Spectra

LACI also provides valuable assessment for patients after Tetralogy of Fallot (TOF) repair. Gunsaulus et al. (43) demonstrated that combining LACI with QRS duration and right ventricular volume index significantly improves arrhythmia prediction accuracy, offering a novel risk assessment approach for long-term follow-up after TOF surgery. In the pediatric domain, Shokeir et al. (44) found significantly elevated LACI in children with isolated mitral valve prolapse who had normal LVEF and only moderate regurgitation (0.35 ± 0.09 vs. normal 0.24 ± 0.06, *P* = 0.002). This suggests LACI can identify impaired left ventricular diastolic function early, providing an earlier reference for determining the optimal timing for surgery. These pediatric findings are presented as developmental reference data, distinct from the adult prognostic evidence synthesized elsewhere in this review. In dilated cardiomyopathy (DCM), Vîjîiac et al. (45) demonstrated that combining LACI with left ventricular global longitudinal strain (LV GLS) significantly improved the predictive accuracy of adverse cardiovascular events (C-index increased from 0.68 to 0.79). This study demonstrated that concurrent assessment of atrioventricular coupling function (LACI) and left ventricular systolic function (LV GLS) provides a more comprehensive reflection of cardiac function status and prognostic risk in DCM patients compared to evaluating a single indicator. In a CMR study of thalassemia patients (*n* = 292) by Meloni et al. (46), *β*-thalassemia patients exhibited significantly elevated LACI and impaired bilateral atrioventricular coupling. Crucially, LACI was unrelated to myocardial iron overload markers (T2) but strongly correlated with myocardial fibrosis (LGE). LGE-positive patients exhibited significantly higher LACI values, and LGE emerged as an independent predictor of LACI. A LACI > 23.6% effectively predicted cardiac complications (AUC = 0.79). This suggests LACI primarily reflects myocardial fibrosis rather than iron burden.

## 4 Discussion

### 4.1 Evidence gaps and research biases

Although the LACI field has developed rapidly, the current evidence system exhibits significant structural biases, including uneven technological pathways, publication bias favoring positive results, and insufficient population representativeness. We acknowledge that recognizing these limitations aims to clarify priority directions for subsequent studies.

#### 4.1.1 Technical source bias

While rapidly accumulating prognostic evidence, recent LACI research exhibits notable technical source bias. CMR, as the originator of the LACI concept and the measurement gold standard, has provided the most robust prognostic evidence. The MESA study, involving thousands of healthy individuals from diverse ethnic backgrounds, established LACI's long-term predictive value for heart failure, atrial fibrillation, and cardiovascular events. This value has been validated in patients with acute myocardial infarction (47), hypertrophic cardiomyopathy (31), and amyloidosis (16). This widespread adoption highlights a mismatch in translational research: while CMR has accumulated a substantial body of evidence on LACI, its routine clinical accessibility is limited by cost, infrastructure, and contraindications; conversely, echocardiography could potentially apply LACI to broad clinical practice but lacks parallel validation under these same conditions. Consequently, the field faces a structural—rather than merely technical—gap between evidence and practice. However, these high-quality studies primarily center on CMR. As a more widely available technology, ultrasound's equivalence to CMR remains insufficiently validated. Consequently, despite strong evidence, translating LACI into clinical practice presents challenges.

Second, CT demonstrates unique value in specific scenarios (24, 48), compared to ultrasound, CT systematically overestimates LACI (6), thereby creating a dangerous translation gap. Without cross-modality calibration, applying CT-based thresholds (24) to ultrasound measurements may lead to misclassification of patients, resulting in either undertreatment of high-risk individuals or overtreatment of low-risk individuals. The absence of such calibration studies directly hinders the safe clinical application of this technology, as clinicians currently lack guidance on how to reconcile numerical discrepancies between different imaging modalities.

Finally, technical heterogeneity within ultrasound examinations constitutes a subtle yet far-reaching source of bias. The vast majority of LACI prognostic studies employ the two-dimensional Simpson method (13, 14, 27, 29), which relies on geometric assumptions of limited accuracy; a small number of studies use RT-3DE (16, 19), a technique that, while offering higher CMR consistency (15), has not yet had its prognostic thresholds validated. A critical question remains: whether risk thresholds derived from 2D ultrasound remain valid when applied to 3D measurements. In the absence of head-to-head prognostic comparisons within the same cohort, clinicians cannot determine whether re-evaluation with 3D ultrasound of 2D ultrasound patients classified as high-risk would alter their risk stratification. This uncertainty may undermine clinical confidence; if physicians cannot be certain that the chosen imaging modality will yield comparable risk stratification results, they cannot safely implement LACI. Future research must prioritize cross-platform calibration within the same patient population to establish equivalence mapping, rather than merely accumulating isolated positive results within a single modality.

In addition to technical heterogeneity itself, there are three sources of variability that warrant particular attention and are often underestimated. These include inter-manufacturer variability; currently, there are no calibration formulas available for LACI values measured across different ultrasound systems (such as Philips, GE, Siemens, and Canon), which undermines the reliability of multicenter or long-term comparisons across different devices. Operator dependency: Although CMR benefits from standardized acquisition protocols and centralized interpretation, LACI in echocardiography remains susceptible to decisions made by bedside operators regarding probe placement, gain settings, and frame selection. This limitation is widely recognized in quantitative echocardiography (17, 49), yet few specific quantitative studies have addressed LACI. Image quality also varies; poor acoustic windows caused by obesity, chronic obstructive pulmonary disease, and chest deformities can reduce endocardial clarity, leading to underestimation of ventricular volume or exclusion of cases. This limitation is rarely reported in published echocardiographic LACI studies. Taken together, these factors reduce the generalizability of single-center, single-operator LACI data to real-world clinical practice, constituting an important but underreported source of technical bias.

#### 4.1.2 Publication bias

Notably, nearly all current LACI prognostic studies report positive outcomes, with negative results or studies showing no incremental value being extremely rare. This distribution pattern suggests potential publication bias in the LACI field and may indicate that LACI's clinical value remains unclear in terms of disease specificity. Does LACI retain significant value in patients with isolated hypertension, mild degenerative valvular disease, or well-controlled type 2 diabetes? Does it possess prognostic value independent of left atrial volume indices? Therefore, identifying clinical scenarios where LACI offers no incremental value is equally important as confirming its advantageous applications. To map the complete scope of LACI applicability, publishing methodologically rigorous studies demonstrating negative results may be encouraged.

#### 4.1.3 Evidence Gap

A critical gap exists in the current evidence chain for LACI. While its prognostic value has been thoroughly validated by multicenter cohort studies, no randomized controlled trials (RCTs) guiding treatment strategies based on LACI have been published to date. Furthermore, basic methodological questions such as the repeatability of 3D LACI measurements and the minimum number of cardiac cycles required under atrial fibrillation remain unresolved. The core question remains: Is LACI improvement a surrogate endpoint for therapeutic efficacy, or a concomitant phenomenon of prognostic enhancement? Specifically, it remains unanswered whether LACI-guided strategies—such as initiating anticoagulation in HCM patients without AF, optimizing pacemaker AV intervals in HFrEF patients, or adjusting diuretic doses in HFpEF patients—outperform conventional approaches. This causal inference question cannot be resolved through observational studies, constituting the primary bottleneck hindering LACI's transition from diagnostic biomarker to clinical therapeutic target. Addressing this gap would elevate LACI from a static risk stratification marker to a functional biomarker capable of dynamically monitoring treatment response and guiding clinical decision-making.

#### 4.1.4 Methodological heterogeneity

Current LACI studies exhibit significant heterogeneity in cutoff values. These cutoff values originate from single-center retrospective analyses, lack external validation, and involve heterogeneous measurement techniques. Without establishing cross-platform calibration methods and refining cutoff validation processes, LACI risks facing threshold confusion similar to that experienced by the left atrial volume index in its early stages, severely hindering guideline adoption. The heterogeneity of cutoff values may be addressed through the following approaches: First, establish linear calibration equations between different echocardiography techniques and manufacturer devices using the MESA study's CMR-LACI as a benchmark, enabling cross-platform comparability of numerical values. Alternatively, for the same target population (e.g., HFpEF, HCM, compare the Net Reclassification Improvement (NRI) and Integrated Discrimination Improvement (IDI) of multiple candidate cutoff values. Alternatively, an Individual Participant Data Meta-analysis (IPD-MA) could integrate raw data from existing cohorts to establish disease-specific and age-, sex-, and disease-specific evidence-based cutoff value recommendations.

Based on the preceding technical analysis and evidence assessment, this paper proposes replicable technical pathway options, quality control systems, and a phased integration roadmap for ultrasound departments implementing LACI assessment.

#### 4.1.5 Advantages over existing diastolic parameters

A key issue in clinical translation is whether LACI can provide information beyond what is covered by existing echocardiographic parameters. Although LAVI reflects the burden of atrial remodeling, it cannot distinguish between physiological adaptation relative to ventricular size and pathological dilation. Although E/e′ is widely used to estimate left ventricular filling pressure, this parameter is highly dependent on loading conditions and operator expertise, and exhibits significant interlaboratory variability, which limits the comparability of data. Although left atrial strain (LA strain) allows for an in-depth assessment of atrial myocardial deformation, it remains a chamber-specific parameter that cannot directly quantify hemodynamic coupling; furthermore, clinical application is hindered by algorithm variations of up to 10%–15% among different manufacturers. LV GLS, on the other hand, has limited sensitivity for isolated diastolic dysfunction and typically remains normal until the late stages of the disease, making it unsuitable for the detection of early HFpEF.

LACI reflects the mechanical interdependence between the atria and ventricles by integrating atrial and ventricular volume data into a single coupling index. Recent studies have shown (21, 27, 29) that LACI retains independent prognostic value even after adjusting for these traditional parameters. These findings emphasize that LACI is not merely a substitute for existing parameters but provides additional pathophysiological information regarding atrioventricular coupling that cannot be obtained from single-chamber indices alone. Its value lies in offering a holistic, ratio-based assessment that reflects the comprehensive hemodynamic status of the left heart, rather than a simple summation of isolated ventricular measurements.

### 4.2 Technical pathway selection strategy

The following recommendations reflect the expert consensus reached by the authors based on the aforementioned technical characteristics and prognostic evidence; however, they have not yet been validated by prospective comparative studies. Similar caveats apply to Sections 4.3 through 4.5.

Based on the clinical scenario, device availability, and operator experience, we propose a tiered approach to LACI assessment technique selection (Table 3). Specifically, the combined STE-LACI assessment is primarily applicable for mechanism exploration and therapeutic target research. Given its time-consuming nature, limited reproducibility, and dependence on image quality, we do not recommend it as a first-line screening tool in routine clinical practice.

**Table 3:** Echocardiographic LACI technical pathway selection and scenario positioning.

Clinical Scenario	Recommended Technique	Key Considerations	Core Limitations
Large-scale screening/epidemiological follow-up	2D Simpson method	5−8 min, accessible, low-cost	Low accuracy, not comparable to 3D/CMR
Precise diagnosis (CMR-correlated, preoperative assessment)	Real-time 3D ultrasound	High accuracy (r = 0.88−0.94), no geometric assumptions	Dependent on image quality, 10–15% failure rate, 10–15 min duration
Mechanism Studies/Therapeutic Target Exploration	3D + Speckle Tracking	Integrated volume and strain assessment	>20 min, research-only
Intraoperative/Point-of-Care Real-Time Assessment	2D or Simplified 3D	Immediate, simple	Low precision, requires labeling as “intraoperative rapid assessment”
Multicenter Clinical Research	3D + AI-assisted	Controllable center effects, high reproducibility	Standardization not established, requires central lab
Exercise Stress Functional Assessment	Stress echocardiography + 2D/3D	Identifies subclinical diastolic dysfunction	Technology immature, few studies, no recommended protocol
Pediatric/Congenital Heart Disease	3D echocardiography	Age-specific reference values	Difficult acquisition, requires experienced operator

**Figure 3:**
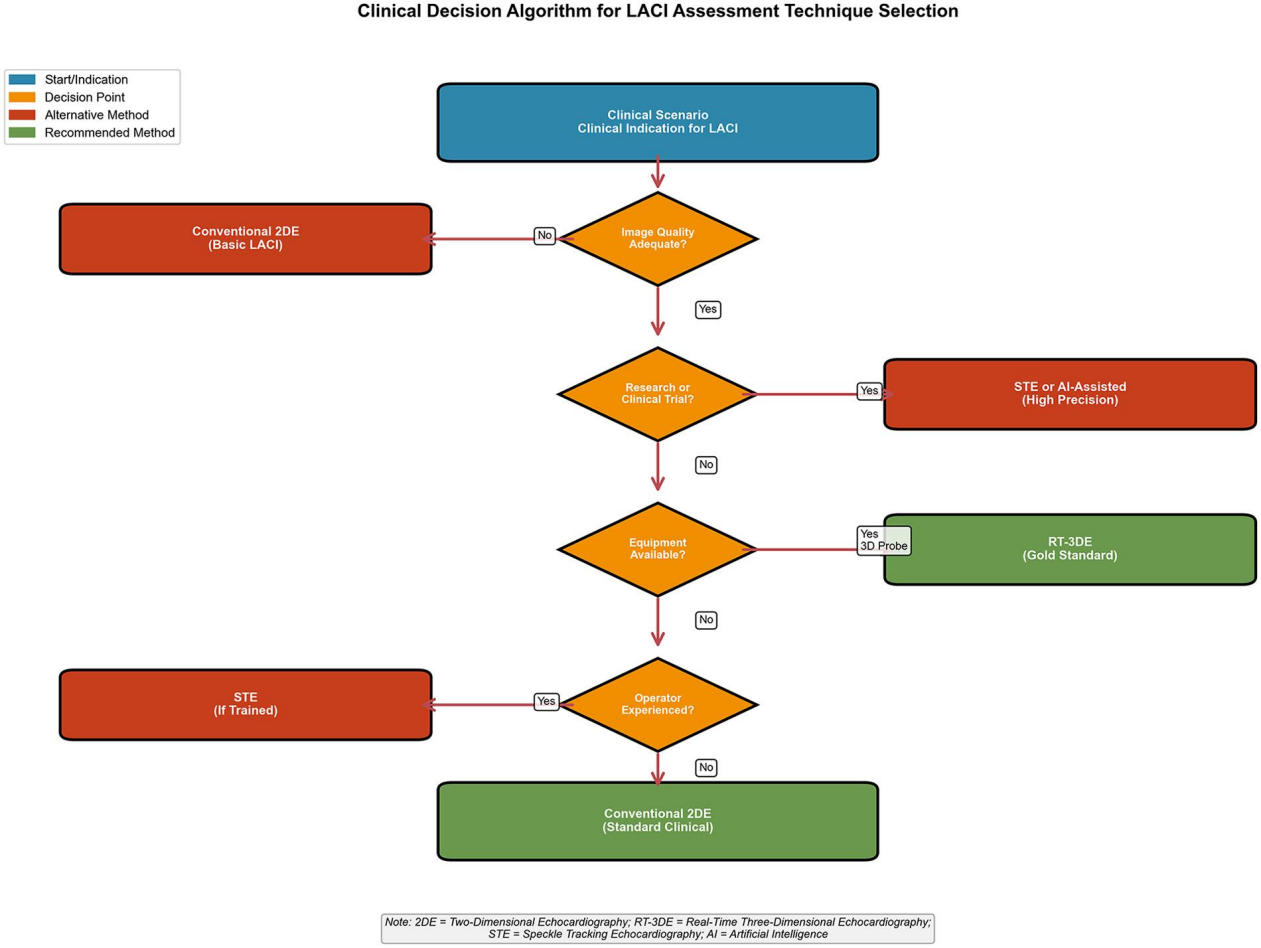
Proposed decision algorithm for selecting LACI measurement technology based on clinical scenario, device availability, and operator experience. Pathways include screening (2D), precise diagnosis (3D), mechanism exploration (3D + STE), and research applications (3D + AI).

Figure 3 presents a decision algorithm for selecting LACI technology based on clinical scenarios, device availability, and operator experience, serving as a reference for routine clinical workflows.

### 4.3 Key quality control points

It should be emphasized that the following quality control recommendations represent the authors’ expert suggestions based on current literature and clinical experience. They should be applied and interpreted with caution, as no prospective studies have yet demonstrated improved outcomes or measurement reliability following strict adherence to these specific protocols.

Standardized protocols must be followed for acquiring echocardiographic images to ensure reproducibility of LACI measurements. During 2D echocardiography, the apical four-chamber and two-chamber views should display the entire left atrium, avoiding shortening or stretching artifacts. Images should be captured at the R-wave peak during end-expiratory phase, with 3–5 consecutive cardiac cycles stored for offline analysis. During real-time 3D echocardiography, acquire full-volume datasets at a frame rate >20 fps, taking care to avoid stitching artifacts during breath-hold. Accurate cardiac identification is also critical for relative LACI calculations. LVEDV should be measured at mitral valve closure, typically corresponding to the QRS peak; end-diastolic left atrial volume is also measured at this point. In cases with ECG conduction disturbances, particularly complete left bundle branch block or right bundle branch block, the QRS complex may be broadened or fragmented, introducing additional variability in QRS peak detection and ECG gating. Under these circumstances, operators should verify phase alignment by combining ECG timing with anatomic landmarks (mitral valve closure click and ventricular septal motion) rather than relying solely on automated QRS detection, and should document any manual adjustments made to phase identification (17). During atrial fibrillation, due to beat-to-beat variability in left atrial filling, LACI measurement requires specific adjustment protocols. A standardized workflow is recommended: (1) Acquire 10–15 consecutive cardiac cycles, discard cycles with poor image quality or incomplete atrial emptying, and retain ≥8 analyzable cycles; (2) Measure LAEDV and LVEDV for each screened cycle, calculating LACI mean, standard deviation (SD), and coefficient of variation [CV = (SD/mean)  ×  100%]; (3) If CV ≤ 15%, data quality is adequate and the mean is reported; if CV > 15%, expand analysis to 20 cycles until CV ≤ 15% or the maximum analyzable cycle count is reached. This protocol is based on Meloni et al.'s (46) cardiac MRI study and complies with the American Society of Echocardiography's guidelines for quantitative cardiac chamber measurements (49).

A complete LACI ultrasound report should include the imaging technique pathway, equipment manufacturer, model, and software version, along with raw volume data (LAEDV, LVEDV) and calculated LACI values. Quality control should assess intra-observer and inter-observer variability. Quarterly analysis of 10% randomly selected cases requires an intraclass correlation coefficient >0.90 and coefficient of variation <8%. Operators must undergo certification; new operators must complete 50 supervised studies and demonstrate required agreement with senior observers before independent operation.

**Figure 4:**
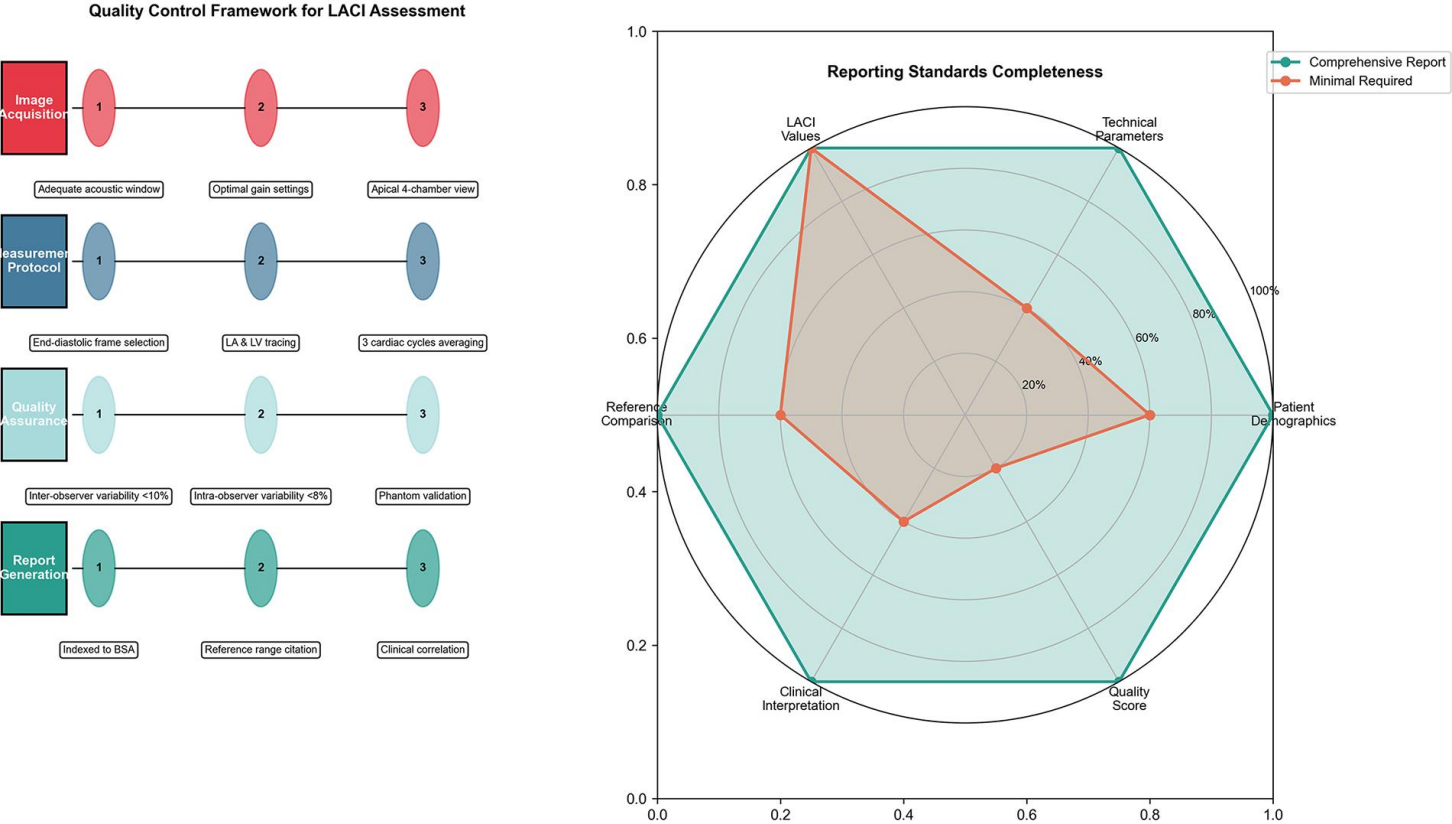
Quality control framework for LACI assessment (left) and structured reporting requirements (right). Blue: comprehensive standards; Orange: minimum requirements.

Figure 4 (left) summarizes the full-process quality control framework for LACI assessment, forming a closed loop from image acquisition to report generation; (right) displays the completeness requirements for LACI structured reports, with blue lines indicating comprehensive reporting standards and orange lines denoting minimum reporting requirements.

### 4.4 Phased clinical integration plan

The phased integration plan outlined below reflects the authors’ proposed roadmap based on expert consensus. These recommendations should be considered tentative and applied with appropriate caution, given the absence of prospective validation demonstrating the feasibility, cost-effectiveness, or clinical benefit of this specific implementation sequence in real-world ultrasound departments.

**Figure 5:**
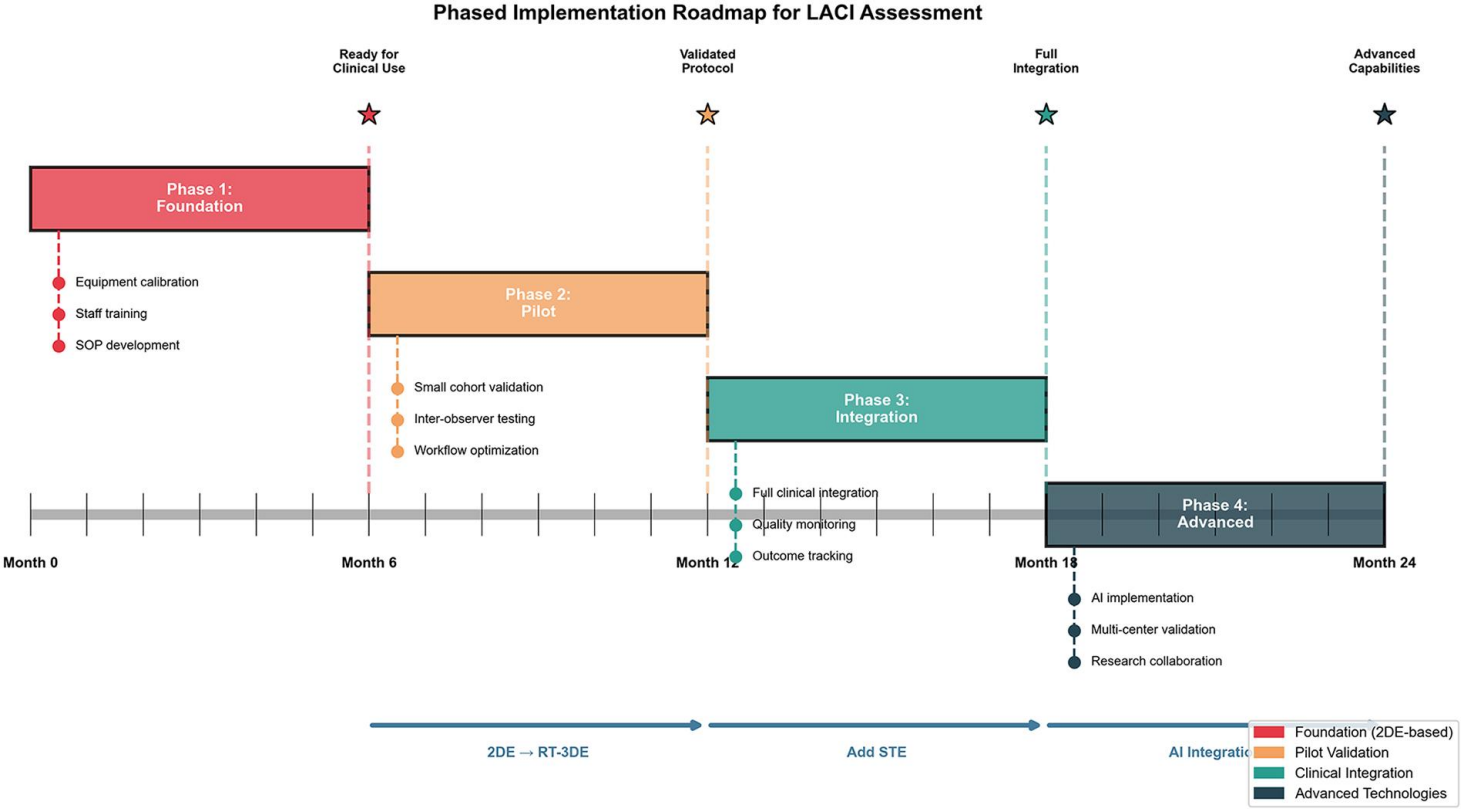
Proposed phased implementation roadmap for LACI clinical integration: infrastructure development (0−6 months), pilot validation (6−18 months), clinical integration (18 + months), and advanced applications.

Figure 5 presents the phased implementation roadmap for transitioning LACI from a research tool to routine clinical practice, encompassing four stages: infrastructure development (0–6 months), pilot validation (6–18 months), clinical integration (post-18 months), and advanced applications. Milestone achievements for each phase are annotated.

Phase 1 should routinely add LACI to high-risk outpatient patients with HFpEF and HCM. Inclusion criteria should be age ≥18 years (reflecting the adult focus of this review; pediatric LACI assessment requires dedicated age-specific protocols and reference systems not covered herein), sinus rhythm, acceptable image quality, and complete apical view. Patients with confounding factors such as moderate-to-severe valvular disease, congenital heart disease, or cardiomyopathy should be excluded. The objective of this phase is to establish standardized departmental procedures through accumulating experience with 50–100 cases, while meticulously documenting the time required for each measurement and image quality scores to establish baseline data for subsequent quality control. Subsequently, Phase II will integrate LACI into the ultrasound reporting system to conduct retrospective association analyses with clinical outcomes such as heart failure, atrial fibrillation, and stroke. This will establish the distribution characteristics of LACI stratified by age and gender. Active participation in multicenter studies will also validate the external applicability of existing cutoff values. Finally, in the third phase, semi-automated or fully automated AI analysis tools should be introduced to reduce individual case analysis time. Participation in randomized controlled trials should explore LACI-guided clinical intervention pathways, driving the integration of LACI into regional healthcare quality control metrics and cardiovascular imaging diagnostic guidelines to achieve its clinical translation.

### 4.5 Echocardiography LACI structured report recommended template

The structured reporting template presented herein represents the authors’ suggested format for standardizing LACI documentation. This template should be regarded as a preliminary proposal rather than an established standard, as no studies have validated whether adoption of this specific reporting format improves communication quality, clinical decision-making, or patient outcomes.

To ensure standardized reporting and facilitate clinical implementation, we recommend adoption of a structured reporting template for echocardiographic LACI assessment (Table 4).

**Table 4:** Recommended structured reporting template for echocardiographic LACI assessment.

Item	Content Specification	Required
Patient Identifier	Patient ID/Examination Date	Yes
Measurement Technique	□ 2D Simpson □ RT-3DE □ Other	Yes
Equipment and Software Information	Manufacturer/Model/Software Version	Yes
Left Atrial End-Diastolic Volume (LAEDV)	XX mL	Yes
Left Ventricular End-Diastolic Volume (LVEDV)	XX mL	Yes
Left Atrial Volume Index (LAVI)	XX mL/m^2^	Recommended
Left Atrioventricular Coupling Index (LACI)	= LAEDV/LVEDV = 0.XX	Yes
Reference Range	Male: <40y < 0.22, 40–59y < 0.25, ≥60y < 0.27; Female: <40y < 0.22, 40–59y < 0.30, ≥60y < 0.32 (7)	Recommended
Conclusion	□ Normal □ Elevated	Yes
Remarks	Atrial fibrillation rhythm, image quality, etc.	As needed

### 4.6 Limitations

This paper focuses on echocardiographic technical pathways, providing only reference descriptions of imaging modalities like CMR and CT without systematically comparing measurement equivalence or prognostic efficacy differences across multimodal imaging techniques. The methodology relies on qualitative analysis and synthesis of existing literature, without employing systematic reviews or meta-analyses to quantitatively summarize LACI's prognostic efficacy.

The vast majority of the included LACI prognostic studies are single-center, retrospective analyses with relatively small sample sizes (Table 2). Of the 14 key prognostic studies summarized, 12 employed a retrospective design, with 11 conducted at a single center; the sample sizes of the echocardiographic studies ranged from 60 to 373 patients, and event rates were often low [for example, only 8 cerebrovascular events were reported in the study by Demir et al. (32)]. These methodological limitations increase the risk of selection bias, confounding factors, and overfitting, and limit the external validity of the reported thresholds. To date, no large-scale, multicenter, prospective echocardiographic cohort studies have been established to validate these findings.

Furthermore, the impact of operator proficiency on LACI measurements remains unquantified, and variations in grassroots practice may compromise measurement reproducibility. Although the proposed quality control system, pathway selection algorithm, and phased implementation roadmap are informed by published evidence on LACI technical validation and prognostic studies (3, 4, 16, 23, 27, 29), their feasibility and cost-effectiveness have not been validated through prospective studies in real-world clinical settings.

Finally, the practical barriers to routine implementation remain underestimated. First are cost barriers, including matrix probes, proprietary software, and lengthy analysis times. Training also requires significant resources, and the software currently lacks vendor-independent automation tools. There are also specific differences among vendors, and a lack of cross-platform calibration. Furthermore, in high-throughput laboratories, the analysis time per case increases by an additional 5 to 15 min, adding to the workflow burden. These limitations suggest that initial implementation should target high-risk subspecialty clinics rather than universal screening.

## 5 Conclusion and future research directions

LACI is a promising marker for assessing diastolic function and atrioventricular coupling, and its prognostic value has been established in HFpEF, HCM, and metabolic diseases. However, technical heterogeneity, fragmented reference ranges, and inconsistent thresholds limit its clinical application.

The limitations of this review include: (i) reliance on predominantly single-center retrospective studies with modest sample sizes; (ii) absence of prospective validation for the proposed quality control protocols and implementation roadmaps; and (iii) lack of systematic cross-modal equivalence testing between echocardiography and CMR/CT.

Future research should prioritize: (1) cross-vendor calibration and device-independent automated algorithms; (2) multicenter prospective studies to establish population-specific reference values; and (3) randomized controlled trials evaluating LACI-guided therapeutic strategies.
